# Reference Values for Liver Stiffness in Newborns by Gestational Age, Sex, and Weight Using Three Different Elastography Methods

**DOI:** 10.3390/jcm14155418

**Published:** 2025-08-01

**Authors:** Ángel Lancharro Zapata, Alejandra Aguado del Hoyo, María del Carmen Sánchez Gómez de Orgaz, Maria del Pilar Pintado Recarte, Pablo González Navarro, Perceval Velosillo González, Carlos Marín Rodríguez, Yolanda Ruíz Martín, Manuel Sanchez-Luna, Miguel A. Ortega, Coral Bravo Arribas, Juan Antonio León Luís

**Affiliations:** 1 Diagnostic Imaging Department, Paediatric Radiology Section, Gregorio Marañón General University Hospital, 28009 Madrid, Spain; angelmaria.lancharro@salud.madrid.org (Á.L.Z.); alejandra.aguado@salud.madrid.org (A.A.d.H.); yolandajose.ruiz@salud.madrid.org (Y.R.M.); 2 Maternal and Infant Research Unit Alonso Family Foundation (UDIMIFFA), Gregorio Marañón Health Research Institute (IiSGM), 28009 Madrid, Spain; msgomezdeorgaz@salud.madrid.org (M.d.C.S.G.d.O.); ppintado@salud.madrid.org (M.d.P.P.R.); pablo.gonzalez@iisgm.com (P.G.N.); perceval.velosillo@iisgm.com (P.V.G.); msluna@salud.madrid.org (M.S.-L.); cbravoarribas@gmail.com (C.B.A.); jaleon@ucm.es (J.A.L.L.); 3 Department of Public and Maternal-Child Health, Faculty of Medicine, Complutense University of Madrid, 28040 Madrid, Spain; 4 Department of Neonatology, Gregorio Marañón General University Hospital, 28009 Madrid, Spain; 5 Department of Obstetrics and Gynecology, Gregorio Marañón General University Hospital, 28009 Madrid, Spain; 6 Department of Medicine and Medical Specialties, Faculty of Medicine and Health Sciences, University of Alcalá, 28801 Alcalá de Henares, Spain; 7 Ramón y Cajal Institute for Health Research (IRYCIS), 28034 Madrid, Spain

**Keywords:** elastography, liver stiffness, newborns, gestational age, reference values, point shear wave elastography (pSWE), two-dimensional shear wave elastography (2D-SWE)

## Abstract

**Objective:** To determine reference values of liver stiffness during the first week of extrauterine life in healthy newborns, according to gestational age, sex, and birth weight, using three elastography techniques: point shear wave elastography (pSWE) and two-dimensional shear wave elastography (2D-SWE) with convex and linear probes. **Materials and Methods:** This was a cross-sectional observational study conducted at a single center on a hospital-based cohort of 287 newborns between 24 and 42 weeks of gestation, admitted between January 2023 and May 2024. Cases with liver disease, significant neonatal morbidity, or technically invalid studies were excluded. Hepatic elastography was performed during the first week of life using pSWE and 2D-SWE with both convex and linear probes. Clinical and technical neonatal variables were recorded. Liver stiffness values were analyzed in relation to gestational age, birth weight, and sex. Linear regression models were applied to assess associations, considering *p*-values < 0.05 as statistically significant. **Results:** After applying exclusion criteria, valid liver stiffness measurements were obtained in 208 cases with pSWE, 224 with 2D-SWE (convex probe), and 222 with 2D-SWE (linear probe). A statistically significant inverse association between liver stiffness and gestational age (*p* < 0.03) was observed across all techniques except for 2D-SWE with the linear probe. Only 2D-SWE with the convex probe showed a significant association with birth weight. No significant differences were observed based on neonatal sex. The 2D-SWE technique with the convex probe demonstrated significantly shorter examination times compared to pSWE (*p* < 0.001). **Conclusions:** Neonatal liver stiffness measured by pSWE and 2D-SWE with a convex probe shows an inverse correlation with gestational age, potentially reflecting the structural and functional maturation of the liver. These techniques are safe, reliable, and provide useful information for distinguishing normal findings in preterm neonates from early hepatic pathology. The values obtained represent a valuable reference for clinical hepatic assessment in the neonatal period.

## 1. Introduction

Ultrasound is a fundamental imaging tool in neonates and pediatric patients for the diagnosis of liver disease, as well as for the follow-up and evaluation of treatment response in certain conditions. It allows for the assessment of liver morphology and echostructure, vascularization, and the detection of malformations or focal lesions [1]. However, conventional ultrasound has limitations in evaluating diffuse liver disease, as such conditions often produce subtle alterations in hepatic echotexture. These changes can be influenced by physiological factors, equipment characteristics, probe selection, and software settings. Moreover, interpretation is highly subjective and largely dependent on the operator’s experience, which limits its diagnostic reliability in this context.

Another classic method for detecting liver pathology is palpation, which primarily helps assess organ enlargement, masses, or abnormal stiffness. However, this approach also heavily depends on the pediatrician’s experience and only allows for a superficial evaluation of the liver [2].

Elastography addresses these limitations by quantifying tissue stiffness, a parameter that necessarily varies with different physiological and pathological mechanisms [2,3].

Image-based elastography, or sonoelastography—integrated into conventional B-mode ultrasound—has become an increasingly useful and widely adopted tool. Its integration allows precise selection of the region of interest, avoiding vascular structures and targeting suspected areas in cases of heterogeneous involvement [2,3].

The use of shear wave elastography (SWE) for non-invasive liver function assessment has grown rapidly. Acoustic Radiation Force Impulse (ARFI)-based techniques, including point shear wave elastography (pSWE) and two-dimensional shear wave elastography (2D-SWE), have been available for nearly a decade [3,4,5,6].

Currently, several ultrasound manufacturers incorporate ARFI technology (both pSWE and 2D-SWE) into their devices, offering recommendations for optimal technique and quality assessment [3,6].

Hepatic elastography is increasingly used in neonates admitted to intermediate or intensive care units—for example, to evaluate cholestatic conditions such as biliary atresia, or diffuse hepatic involvement of infectious, metabolic, or storage origin [7,8].

However, to date, no publication has addressed the specific considerations for bedside elastography in infants within neonatal intensive care units (NICUs) or intermediate care units (ICUs).

Ultrasound and its complementary tools can be extremely valuable in the initial management and monitoring of these conditions. Furthermore, an ultrasound parameter that is sensitive to gestational age at birth may be key to understanding hepatic maturation, anticipating clinical risks, and personalizing neonatal care. Validating such a parameter could benefit both clinical practice and perinatal research.

The objective of this study was to determine gestational age-specific reference values for liver stiffness using sonoelastography in the first week of extrauterine life, based on various birth-related parameters.

## 2. Materials and Methods

### 2.1. Study Design

We conducted a cross-sectional observational study on a hospital-based cohort of healthy newborns (NBs) between 24 + 0 and 42 weeks of gestation, admitted to our center between January 2023 and May 2024. After meeting inclusion criteria, liver elastography was performed within the first week of postnatal life. The study was approved by the Ethics Committee for Research with Medicines of Hospital Gregorio Marañón (CEIm EHP 92/23) on 22 May 2023.

Following the methodology described by Royston and Wright [9] for establishing reference values, at least 15 subjects were recruited per gestational age week, or until the study period was completed. If the minimum number was not met, cases were grouped into clinically relevant gestational age intervals (≤28 weeks, 29–32 weeks, 33–36 weeks, 37–39 weeks, and ≥40 weeks). Gestational age was determined in all cases via ultrasound performed between the 9th and 13th week of gestation.

### 2.2. Inclusion Criteria

Recruitment was carried out with the support of the research team members from the Radiology, Obstetrics, and Neonatology departments. Inclusion criteria consisted of the absence of structural fetal malformations and/or known chromosomal or genetic anomalies, no evidence of congenital infection, and no prenatal diagnosis of fetal growth restriction as defined by International Society of Ultrasound in Obstetrics and Gynecology (ISUOG) criteria [10]. In all cases, informed consent was obtained from the parents or legal guardians.

### 2.3. Exclusion Criteria

Cases with early perinatal death, significant neonatal morbidity (pulmonary hypertension of cardiac or pulmonary origin), or evidence or development of liver pathology (hypoxic, cardiovascular, or cholestatic) were excluded since, as described in the literature (Li et al., 2020) [11], these could be factors that elevate the data collected on liver stiffness studied by elastography.”. Also excluded were technically invalid studies (mean and median RMI < 0.4 for all techniques and probes, or IQR/Med > 30% for kPa and >20% for m/s across all methods) and cases in which parents declined participation.

### 2.4. Collected Anthropometric and Clinical Variables

All included newborns underwent hepatic sonoelastography, described in detail below.

Clinical maternal variables included type of gestation (singleton vs. twin) and mode of delivery (spontaneous vaginal, assisted vaginal, cesarean). Neonatal anthropometric variables included sex (male vs. female), gestational age (weeks and days), birth weight (grams), length (cm), head circumference (cm), and percentiles of weight, length, and head circumference using the 2013 Fenton growth charts [12] and the clinical tool PediTools [13].

Other neonatal clinical variables included early neonatal mortality, presence or absence of neonatal respiratory distress, perinatal asphyxia, neonatal hipoxic-isquemic injury, common intra- or extracardiac shunts (patent ductus arteriosus and patent foramen ovale), congenital heart disease, pulmonary hypertension, fetal growth restriction, twin-to-twin transfusion syndrome, and physiological neonatal jaundice.

### 2.5. Technical Variables Collected

#### 2.5.1. B-Mode Ultrasound Technical Variables

During baseline hepatic B-mode ultrasound, the following were recorded: whether the infant was fasting, whether the hepatic parenchymal echo pattern was normal or abnormal (and description, if applicable), and whether the bile ducts and gallbladder appeared normal or abnormal (with corresponding description).

#### 2.5.2. Hepatic Elastography Technical Variables

Point shear wave (pSWE) with convex probe (1–6 MHz) and 2D shear wave (2D-SWE) with convex (1–6 MHz) and linear probes (12–14 MHz).

All scans were performed by two expert radiologists with 20 years of experience in neonatal radiology and 10 years in elastography, who developed a protocol based on previous studies [14,15,16]. The study included patients admitted to NICU or intermediate care units during the study period who met the inclusion criteria described in Section 2.2. Technical aspects of scanning in this population have been previously described by our group [17].

In addition to routine hepatic ultrasound, three elastography modalities were performed using a Samsung RS85 ultrasound system: pSWE with a convex 1–6 MHz probe, 2D-SWE with the same convex probe, and 2D-SWE with a 12–14 MHz linear probe. Liver stiffness was recorded in both kilopascals (kPa) and meters per second (m/s). Variables created included: kilopascals by point shear wave elastography (pSWE_Kpa), meters per second by point shear wave elastography (pSWE_ms), kilopascals by two-dimensional shear wave elastography with convex probe (2DSWEcx_kPa), meters per second by two-dimensional shear wave elastography with convex probe (2DSWEcx_ms), kilopascals by two-dimensional shear wave elastography with linear probe (2DSWEln_kPa), and meters per second by two-dimensional shear wave elastography with linear probe 2DSWEln_ms. Additional safety parameters included scan duration (minutes), mechanical index, and thermal index.

Probes were placed transversely in a subcostal position, right parasagittal midline, targeting hepatic segment IV, as shown in Lancharro Zapata et al. [17]. For pSWE, a minimum ROI (1 cm^2^) was placed at least 1.5 cm below the liver capsule and up to 8 cm from the skin surface. Measurements were valid if at least 10 readings had an RMI > 0.4 (Samsung RS85 standard).

For 2D-SWE (both probes), the elastogram with the fewest artifacts was selected. A small ROI was applied (0.3 cm diameter for convex, 0.2 cm for linear), and 10–15 valid measurements were recorded following the same quality criteria.

The reported value of liver stiffness represents the arithmetic median of multiple independent measurements, performed in different areas of the liver parenchyma and at varying depths, to enhance the representativeness and reliability of the final result. This practice reduces variability due to tissue heterogeneity, technical artifacts, or sampling errors. It is a recommended standard to ensure measurement validity, especially in situations where liver stiffness may be influenced by factors other than fibrosis, such as venous congestion secondary to right heart overload or pulmonary hypertension (Dietrich et al., 2017) [2]. Valid averages required an interquartile range/median (IQR/Med) ratio < 30% for kPa and <20% for m/s, per current recommendations [2].

### 2.6. Statistical Analysis

Data were collected using a structured Excel form (Microsoft Corp., Redmond, WA, United States). Statistical analyses were performed using SPSS v26 (IBM Corp., Armonk, NY, United States) and R 4.5.0 (R Foundation for Statistical Computing, Viena, Austria).

Qualitative variables were expressed as absolute and relative frequencies. For quantitative variables, measures of central tendency (mean, median) and dispersion (standard deviation, interquartile range, minimum and maximum) were calculated. These are shown in Table 1.

Reference values were determined using simple linear regression models, with each elastography parameter as the dependent variable and neonatal variables (gestational age in days, birth weight, and sex) as independent variables.

A *p*-value < 0.05 was considered statistically significant.

## 3. Results

### 3.1. Study Population

During the study period, a total of 287 patients were included. Table 1 summarizes the clinical characteristics of the cohort, including gestational, anthropometric, neonatal, and ultrasound variables. Qualitative variables are reported in absolute and relative frequencies, while quantitative variables are expressed as median and standard deviation. The mean gestational age (GA) of the patients was 34.91 weeks (SD ± 4.35). There was a significantly higher proportion of male newborns (60.63% vs. 39.37%).

For the analysis of reference values and after applying exclusion criteria, we obtained a final sample of 199 subjects for quantitative liver elastography using the pSWE technique, 212 for the values obtained using the 2D-SWE technique with a convex probe, and 210 for the values obtained using the 2D-SWE technique with a linear probe. Regarding technical quality, no statistically significant differences were found in the exclusion rates based on the technique used (*p* > 0.05), although the mean examination time was significantly shorter for the 2D-SWE techniques using both probes (*p* < 0.001).

### 3.2. Reference Values of Liver Stiffness by Gestational Age, Birth Weight, and Sex

#### 3.2.1. Gestational Age vs. Liver Stiffness

**Figure 1:**
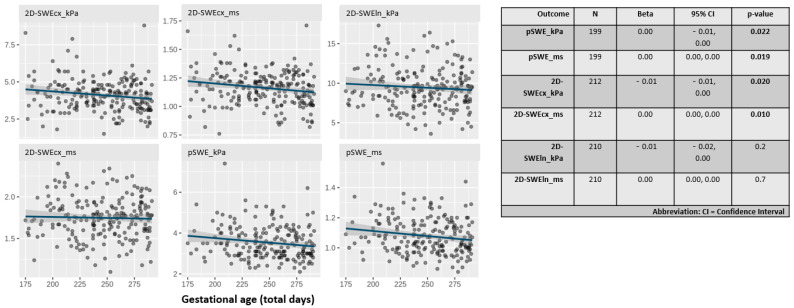
Scatter plots showing the distribution of liver stiffness values in kilopascals (kPa) and meters per second (m/s) using all techniques (pSWE and 2D-SWE) and probes (cx: convex, ln: linear).

Figure 1 displays the distribution of liver stiffness parameters by gestational age in days. All parameters, except those obtained with the linear probe, showed a statistically significant inverse relationship with gestational age (*p* < 0.03).

Notably, liver stiffness measured in kPa using the 2D-SWE technique with the convex probe showed the steepest slope among all techniques.

Table 2 shows the parametrized values of the study variables based on five clinically relevant gestational age intervals. Higher stiffness values were observed in the preterm groups.

#### 3.2.2. Relationship Between Birth Weight and Sex vs. Liver Stiffness

Table 3 indicates that no statistically significant differences were found in any of the parameters studied regarding birth weight (*p* < 0.03).

**Figure 2:**
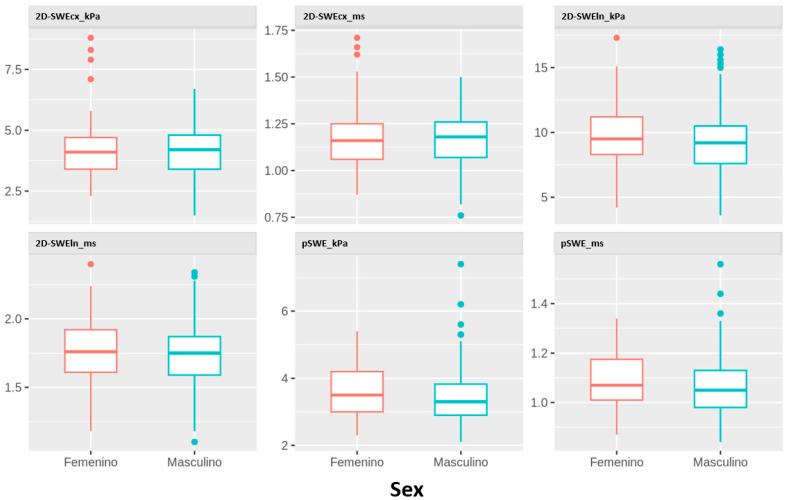
Distribution of kPa and m/s values obtained using all techniques and probes according to sex.

Figure 2 displays the distribution of liver stiffness parameters by sex. No statistically significant differences were found in any of the parameters studied.

## 4. Discussion

The results in 199 newborns (GA 24–41 weeks) show that liver stiffness measured using ARFI-type sonoelastography—both pSWE and 2D-SWE with a convex probe—significantly decreases with each day of gestational age. No significant differences were observed by birth weight and sex.

To date, this is the first study to describe normal reference values for all these parameters in newborns during their first week of life by gestational age at birth, in a single center and using two different techniques and two probe types. Previous studies have analyzed some of these parameters. Specifically, the group led by Fontanilla et al. studied normal liver stiffness using pSWE with convex and linear 10–5 MHz probes in children aged 0–18 years, including 15 children aged 1 day to 1 month. They reported a mean of 1.05 ± 0.04 m/s for this age group using a convex probe and a machine from the same manufacturer as ours. Their results are similar to our findings in the ≥40-week GA group (Table 2), although they did not specify the gestational age of the neonatal group [14]. The study by Galina et al. used the 2D-SWE technique with a single probe in 37 children under 2 years of age. In this group, they reported a median liver stiffness of 3.4 kPa, also similar to our ≥40-week GA group (Table 2), but again, they did not specify how many were neonates or their gestational age [15]. Finally, the group led by Kemmotsu et al. studied liver stiffness in 49 neonates using a convex probe and 2D-SWE, but they did not distinguish between physiological and pathological conditions that may affect liver stiffness in neonates, which likely explains the discrepancy between their results (mean 1.22 m/s) and ours [18].

Regarding the observed trend, as we have described, liver stiffness is greater at lower gestational ages. For the kPa parameter measured by 2D-SWE with a convex probe, our findings are the opposite of what was reported in the study on the fetal liver during intrauterine life by Nalet et al. [19], possibly due to circulatory and metabolic differences between the fetal and neonatal liver. The fetal liver plays a crucial role in hematopoiesis, glycogen storage, and carbohydrate and lipid metabolism. During intrauterine life, it receives approximately 50% of umbilical blood flow. After birth, the neonatal liver relies on portal and arterial blood supply, undergoes internal circulatory reorganization, and takes on critical postnatal functions such as gluconeogenesis, bilirubin metabolism, and protein–lipid regulation—essential adaptations for the newborn’s metabolic and physiological transition [20].

The increased stiffness at lower gestational ages observed in our study may be due to the immature liver in preterm infants, characterized by relatively sparse bile ducts, predominant extramedullary hematopoiesis, underdeveloped hepatic sinusoids, and lower enzymatic activity. These features are more pronounced in extremely preterm infants, who show greater structural and functional immaturity, making them more susceptible to cholestasis, steatosis, and early portal fibrosis, especially under metabolic stress or prolonged parenteral nutrition [21]. Similar findings have been documented in animal models such as preterm baboons, including Kupffer cell hypertrophy, hemosiderosis, and ductal proliferation [22]. Ultrasound findings also show that the preterm liver can be more echogenic, with a larger liver-to-body ratio, accounting for 5–6% of total body volume in preterm neonates [23]. However, in the study conducted by Kemmotsu et al. [18], no significant associations were found with gestational age or birth weight. This may be due to differences in sample size and clinical characteristics, as previously discussed.

As for birth weight, this variable had a lesser impact on liver stiffness. Alison et al. [24] in 63 preterm newborns (<36 weeks GA) using a 6 MHz linear probe and the SuperSonic technique found that lower birth weight percentiles were associated with higher liver stiffness. The group led by Knebelmann et al. [25] studied liver stiffness in 171 healthy children aged 0–18 years using both convex and linear probes with 2D-SWE, but they did not specify how many neonates were included or their gestational age, and found no association between weight and stiffness. Nevertheless, from a perinatal perspective, gestational age is a better predictor than weight for hepatic and multisystemic physiology. While both gestational age and birth weight are important, the evidence suggests that gestational age is a stronger predictor of perinatal morbidity and mortality because it more accurately reflects physiological maturity, which is essential for neonatal survival and development [26,27,28].

Our study found no differences in liver stiffness based on neonatal sex, consistent with other groups [14,15,29]. However, Li et al. reported a slight difference, not statistically significant, in males over 12 years of age, attributed to hormonal and metabolic changes during adolescence [30]. It is worth noting the higher relative proportion of males in our sample, which is consistent with their increased rate of NICU admissions, as supported by various recent studies [11,31].

It is also important to highlight the significantly elevated liver stiffness values in both kPa and m/s when using the linear 12–14 MHz probe with the 2D-SWE technique. These values differ significantly from those obtained with the other two techniques. Postek et al. [16] studied liver stiffness in 58 healthy full-term neonates (GA 38–41 weeks) between days 2 and 28 of life using 2D-SWE and a 10 MHz linear probe, following a fasting period of at least one hour. They reported median values of 1.41 m/s in the right hepatic lobe (RHL) and 1.40 m/s in the left hepatic lobe. These results differ from ours (median of 1.75 m/s with the 12–14 MHz linear probe). We did not find significant differences across groups. Our probe was different, our ROI for measurements smaller, and our sample size larger. Differences are also seen when comparing with Fontanilla et al.’s [14], pSWE results using a 5–10 MHz linear probe, or with Li et al.’s results using a linear L11-3U probe and 2D-SWE, although their study included only 40 children under 1 year of age without specifying the number of neonates or their gestational age. It is likely that in our study, the size and weight of the probe exerted enough surface pressure on the soft abdominal wall of neonates to artifactually increase stiffness values. Our previous study [17] suggested some tips to avoid this, but further research is still needed to confirm this hypothesis. At present, based on our data, we cannot recommend the use of these linear probes in this type of ultrasound machine for neonatal liver studies in NICU or intermediate care settings.

Finally, although technical quality and reliability were similar between pSWE and 2D-SWE using the convex probe, 2D-SWE allowed significantly shorter exam times, which can be advantageous in clinical settings where time is critical.

Despite the number of neonates included, we were unable to achieve a minimum of 15 cases per gestational age during the study period in a single center, which limits the statistical power to establish precise reference values, especially in extremely preterm groups. We recommend validating our findings in other hospitals. These limitations may be due to factors such as high vulnerability, ethical and logistical complexities [32], as well as technical aspects already discussed by our team in a previous study [17].

Additionally, although several techniques and probes were used to evaluate liver stiffness and its relationship with gestational age, birth weight, and sex, we did not assess pSWE using a linear probe. However, the parameters obtained with 2D-SWE and a linear probe in our study did not show a clear correlation with gestational age (unlike the other techniques) nor did they match previously published data. More research is needed to determine the suitability of using this type of probe with 2D-SWE for neonatal liver assessment.

## 5. Conclusions

Gestational age is significantly correlated with liver stiffness parameters at birth, as assessed by point shear wave elastography (pSWE) and two-dimensional shear wave elastography (2D-SWE) using a conventional convex probe.

This technique, as a complementary tool in neonatal liver assessment, is safe, reliable, and provides clinically relevant information. On the one hand, it may reflect the structural and functional maturity of the liver. On the other, it is useful for distinguishing normal findings in early gestational ages from clinical alterations that affect liver function, such as various types of cholestasis, hypoxic–ischemic injury, congestive hepatopathy or intrauterine growth restriction.

## Figures and Tables

**Table 1:** Distribution of maternal and neonatal clinical and ultrasound variables of the study (*n* = 287). pSWE: point shear-wave elastography; 2D-SWEcx: two-dimensional shear-wave elastography by convex probe; 2D-SWEln: two-dimensional shear-wave elastography by linear probe.

Maternal Variables		
**Type of gestation**		
	Single	281 (97.91%)
	Twins	6 (2.09%)
**Type of delivery**		
	Eutocic vaginal	146 (50.87%)
	Instrumented vaginal	29 (10.10%)
	Cesarean section	112 (39.02%)
**Anthropometric measures**		
	Female	113 (39.37%)
	Male	174 (60.63%)
**Gestational age (weeks)**	34.91 (±4.35)	
**Gestational age (total days)**	247.28 (±30.45)	
**Birth weight (gr.)**	2371.42 (±911.09)	
**Birth weight percentile**	39.84 (±28.34)	
**Length (cm.)**	45.12 (±5.75)	
**Length percentile**	46.05 (±29.41)	
**Head circumference (cm.)**	31.81 (±4.30)	
**Head circumference percentile**	46.93 (±30.76)	
**Size at birth**		
	Normal	217 (75.61%)
	Big	20 (6.97%)
	Small	50 (17.42%)
**Neonatal variables**		
**Neonatal respiratory distress**	135 (47.04%)	
**Perinatal asphyxia**	26 (9.06%)	
**Neonatal hypoxic-isquemic injury**	12 (4.18%)	
**Patent ductus arteriosus**	22 (7.67%)	
**Patent foramen ovale and other intracardiac shunts**	36 (12.54%)	
**Congenital heart disease**	13 (4.53%)	
**Pulmonary hipertension**	18 (6.27%)	
**Fetal growth restriction**	29 (10.10%)	
**Twin-to-twin transfusion syndrome**	6 (2.09%)	
**Physiological neonatal jaundice**	81 (22.8%)	
**Ecographic variables**		
**Fasting**	25(8.71%)	
**Pathological liver ultrasound**	21 (7.32%)	
**Pathological biliary tract and gallbladder ultrasound**	0 (0.00%)	
**Examination time (min.)**		
	pSWE	2.28 (±1.71)
	2D-SWEcx	0.70 (±0.45)
	2D-SWEln	0.56 (±0.29)
**Mechanic index**		
	pSWE	1.52 (±0.81)
	2D-SWEcx	1.48 (±0.09)
	2D-SWEln	1.380 (±0.049)
**Thermic index**		
	pSWE	0.28 (±0.13)
	2D-SWEcx	1.03 (±0.16)
	2D-SWEln	0.81 (±0.13)

**Table 2:** Median, mean, standard deviation (SD), minimum (Min.), and maximum (Max.) values of kPa and m/s using pSWE and 2D-SWE with a convex probe, grouped by gestational age intervals in weeks.

Characteristic	<=28 N = 25	29–32 N = 43	33–36 N = 60	37–39 N = 49	>=40 N = 39
**pSWE_kPa**					
**Median (Q1, Q3)**	3.50 (3.10, 4.10)	3.80 (3.00, 4.30)	3.35 (3.00, 4.00)	3.30 (2.80, 3.95)	3.10 (3.00, 3.60)
**Mean (SD)**	3.69 (0.82)	3.84 (0.94)	3.51 (0.75)	3.40 (0.73)	3.37 (0.78)
**Min, Max**	2.60, 5.40	2.50, 7.40	2.30, 5.30	2.10, 5.30	2.30, 6.20
**pSWE_ms**					
**Median (Q1, Q3)**	1.08 (1.03, 1.17)	1.12 (1.00, 1.20)	1.06 (0.99, 1.15)	1.05 (0.97, 1.15)	1.03 (1.00, 1.10)
**Mean (SD)**	1.10 (0.12)	1.12 (0.13)	1.08 (0.11)	1.06 (0.12)	1.06 (0.11)
**Min, Max**	0.92, 1.34	0.91, 1.56	0.88, 1.33	0.84, 1.33	0.87, 1.44
**2D-SWEcx_kPa**					
**Median (Q1, Q3)**	4.20 (3.00, 5.00)	4.40 (3.60, 5.00)	4.10 (3.60, 4.50)	4.20 (3.50, 4.90)	3.60 (3.10, 4.20)
**Mean (SD)**	4.25 (1.40)	4.43 (1.19)	4.01 (0.81)	4.10 (0.95)	3.72 (1.19)
**Min, Max**	2.00, 8.30	1.80, 7.90	1.50, 5.70	2.20, 6.10	2.00, 8.80
**2D-SWEcx_ms**					
**Median (Q1, Q3)**	1.29 (1.06, 1.30)	1.21 (1.09, 1.30)	1.17 (1.09, 1.22)	1.18 (1.07, 1.29)	1.09(1.01, 1.18)
**Mean (SD)**	1.19 (0.19)	1.21 (0.16)	1.16 (0.11)	1.17 (0.14)	1.10 (0.16)
**Min, Max**	0.82, 1.66	0.76, 1.62	0.86, 1.38	0.84, 1.42	0.82, 1.71

**Table 3:** Distribution of kPa and m/s values using all techniques and probes according to birth weight in grams.

Outcome	N	Beta	95% CI	*p*-Value
**pSWE_kPa**	199	−0.01	−0.03, 0.00	0.074
**pSWE_ms**	199	0.00	0.00, 0.00	0.067
**2D-SWEcx_kPa**	212	−0.01	−0.03, 0.00	0.084
**2D-SWEcx_ms**	212	0.00	−0.01, 0.00	0.049
**2D-SWEln_kPa**	210	−0.02	−0.06, 0.01	0.2
**2d-SWEln_ms**	210	0.00	−0.01, 0.00	0.4
**Abbreviation: CI = Confidence Interval**				

## Data Availability

The data used to support the findings of the present study are available from the corresponding author upon request.
